# Host-Guest Inclusion Complexes of Natural Products and Nanosystems: Applications in the Development of Repellents

**DOI:** 10.3390/molecules27082519

**Published:** 2022-04-14

**Authors:** Gueive Astur Pena, Anna Sylmara da Costa Lopes, Sylvano Heleno Salgado de Morais, Lidiane Diniz do Nascimento, Fábio Rogério Rodrigues dos Santos, Kauê Santana da Costa, Cláudio Nahum Alves, Jerônimo Lameira

**Affiliations:** 1 Laboratório de Planejamento e Desenvolvimento de Fármacos, Federal University of Pará, Augusto Correa Street, w/n, Guamá, Belém 66075-110, Brazil; gueive.pena@ifpa.edu.br (G.A.P.); nahum@ufpa.br (C.N.A.); 2 Laboratório de Catalálise e Oleoquímica, Federal University of Pará, Augusto Correa Street, w/n, Guamá, Belém 66075-110, Brazil; annalopes.ta.qi@hotmail.com; 3 Laboratório de Química Analítica e Ambiental, Federal University of Pará, Augusto Correa Street, w/n, Guamá, Belém 66075-110, Brazil; profmoraisq@gmail.com; 4 Museu Paraense Emilio Goeldi, Laboratório Adolpho Ducke, Perimetral Avenue, Nuber 1901, Belém 66077-830, Brazil; lidianenascimento@museu-goeldi.br; 5 Instituto de Ciências da Educação, Universidade Federal do Oeste do Pará, Vera Paz Street, w/n Salé, Santarém 68040-255, Brazil; fabio.santos@ufopa.edu.br; 6 Laboratório de Simulação Computacional, Instituto de Biodiversidade, Universidade Federal do Oeste do Pará, Vera Paz Street, w/n Salé, Santarém 68040-255, Brazil

**Keywords:** nanosystems, essential oils, natural repellents, nanoencapsulation, controlled release, intermolecular interactions

## Abstract

Repellents are compounds that prevent direct contact between the hosts and the arthropods that are vectors of diseases. Several studies have described the repellent activities of natural compounds obtained from essential oils. In addition, these chemical constituents have been pointed out as alternatives to conventional synthetic repellents due to their interesting residual protection and low toxicity to the environment. However, these compounds have been reported with short shelf life, in part, due to their volatile nature. Nanoencapsulation provides protection, stability, conservation, and controlled release for several compounds. Here, we review the most commonly used polymeric/lipid nanosystems applied in the encapsulation of small organic molecules obtained from essential oils that possess repellent activity, and we also explore the theoretical aspects related to the intermolecular interactions, thermal stability, and controlled release of the nanoencapsulated bioactive compounds.

## 1. Introduction

### Repellents: Mode of Action and Current State of Art

Arthropods are vectors of numerous zoonotic diseases caused by viruses and protozoa. The incidence of these diseases has increased significantly worldwide due to the development of resistance of these vectors against commercially available insecticides and repellents. In addition, changes in the environmental conditions, caused by urbanization, overcrowding, and water/soil pollution have contributed to the proliferation of arthropod populations which have led to the spread of the infectious diseases transmitted by these vectors [1].

Repellents are chemical substances that prevent direct contact between the hosts and the arthropods that are vectors of human diseases, acting against the olfactory recognition system of these vectors [2]. Mosquitoes are the main agents of vector-borne diseases in tropical regions of the world, and their olfactory recognition system is complex and involves different recognition proteins involved with the binding to environmental chemical signals. The chemical recognition initiated when odorant molecules present into the environment is recognized by sensory structures, such as the maxillary palps or the sensilla present in the antennae [3].

The olfactory system of mosquitoes includes a wide range of transmembrane receptors located on olfactory neurons that are expressed in different parts of the body of these insects, especially in sensory regions [4]. These receptors evolved to perform various functions during the insect life cycle such as identification of pheromones for reproduction and detection of chemical signals for recognition of hosts [4,5,6]. The odorant-binding proteins (OBPs) are the main recognition olfactory proteins secreted by accessory cells, and these structures are responsible for transporting odorant molecules to neurons involved in olfactory recognition [7]. Structural analyses performed in OBPs have shown similar results related to the recognition of these volatile compounds, especially pheromones [8,9]. Some studies suggest that OBPs interact with sensory neuron membrane proteins (SNMP) and transport pheromones and other aromatic compounds. Similarly, it has been shown that SNMP proteins are necessary for sensitivity to 11-*cis*-vacenyl acetate (cVA) pheromone related to different sexual behaviors in *Drosophila* sp. [10].

The *N*,*N*-diethyl-meta-toluamide (DEET) is the most used synthetic repellent, and its main mode of action is related to the inhibition of OBP1 of insects. DEET shows an effective residual protection against a wide variety of insects and other arthropods [11,12,13,14], however, it can be absorbed through the skin, causing adverse effects on human health [15]. Furthermore, studies have reported the insensitivity of some insects to the application of DEET, which reduces its effectiveness in the repellency activity leading to the need for reapplications [12,16,17,18].

**Figure 1:**
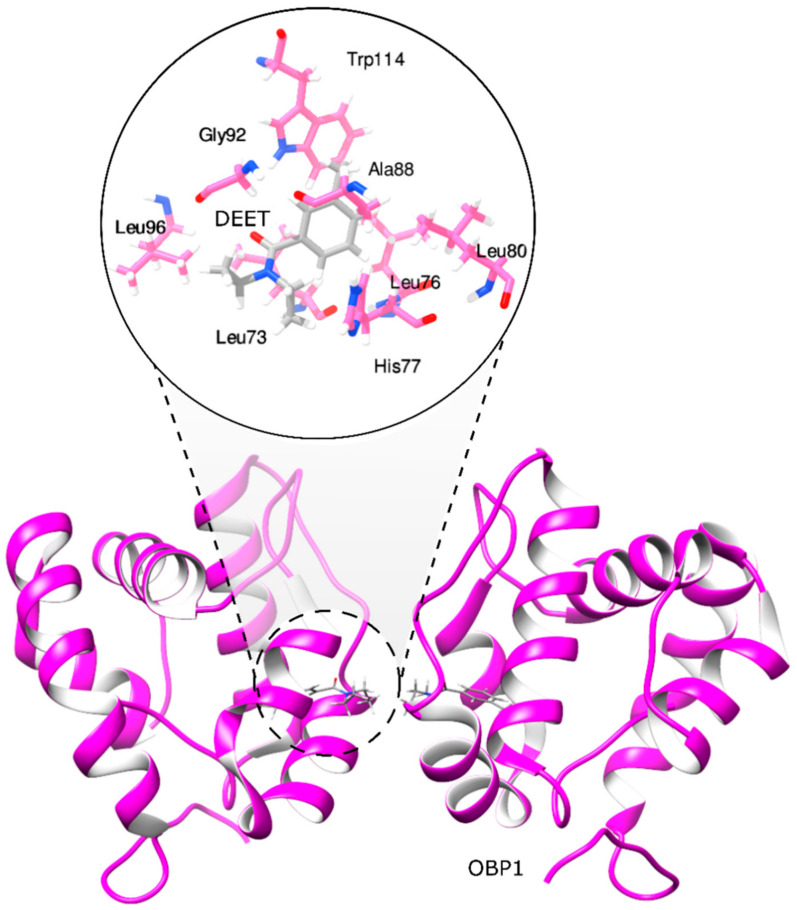
Molecular interactions between odorant-binding protein 1 and DEET.

Different studies have used the mosquito olfactory recognition system, including the OBP1 structure to screen compound libraries of natural and synthetic origins, combining in silico and in vitro approaches [19,20,21]. Currently, different approaches involving the use of synthetic chemistry, molecular modeling, and cheminformatics have been applied in the design and discovery of new bio-inspired repellents using as a start point the structural information obtained from the mode of action of DEET complexed to the OBP1 (Figure 1) [19,21,22]. Based on these approaches, new chemical groups with similar activity to DEET have been identified, such as monoterpenes, amides, piperidines, diols, and phthalates [21,23].

Interest in the development of new repellents using natural products has increased in recent decades, due to their ecologically friendly and biodegradable characteristics [16,24]. Natural products, including essential oils, resins, and substances derived from plants have been applied as repellents by humankind due to their repellent activities since ancient times [25]. Several studies have described the repellent activities of plant derivatives, and essential oils have been pointed to as alternatives to conventional synthetic repellents due to their interesting residual protection, accessibility, and low toxicity to the environment [26].

**Figure 2:**
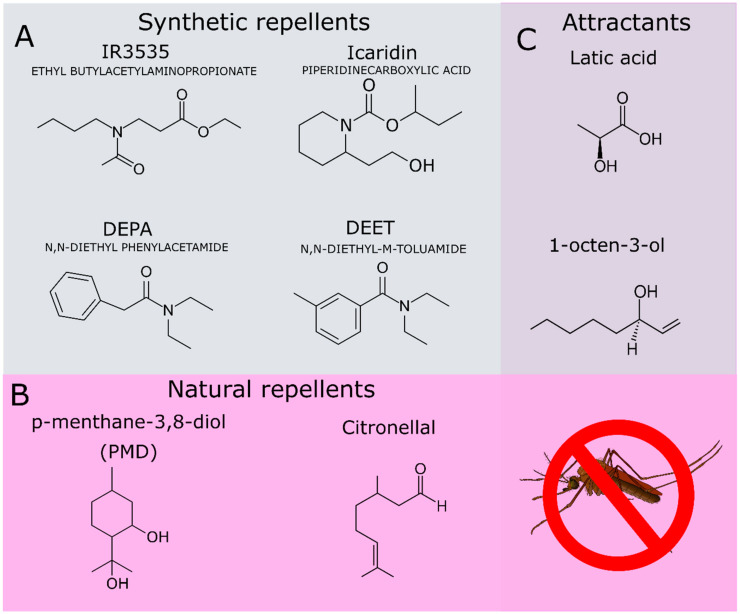
(**A**) Synthetic compounds used as repellents. (**B**) Natural compounds used as repellents. (**C**) Attractant compounds produced by mammals and recognized by the mosquito olfactory system.

In addition to DEET, other repellents of synthetic origin have been applied in commercial formulations, such as picaridin (icaridin, hydroxy-ethyl isobutyl piperidine carboxylate, Figure 2A) and *N*-acetyl-*n*-butyl-3-amino propionate (IR3535, Figure 2A). Currently, several natural compounds from essential oils have been also used in commercial repellent formulations due to their satisfactory repellent activity, such as the p-menthane-3, 8-diol (PMD) [18,27] (Figure 2B) and citronellal (Figure 2B) [28,29]. Some repellents act as competitive inhibitors of attractant molecules that are exhaled by the mammalian hosts. These attractant molecules include L-lactic acid and the 1-octen-3-ol (Figure 2C) [30,31].

**Figure 3:**
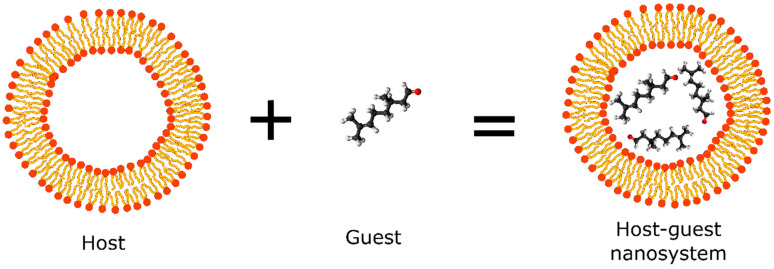
Schematic representation of host-guest nanosystem applied in the development of repellents.

New technologies applied in natural repellent formulations such as the nanoencapsulation of compounds using organic matrices have shown to be a promising area of academic research. Cyclodextrins, polymeric micelles, solid lipid nanoparticles (SLNs), and liposomes are some of the most widely used polymeric systems applied in the nanoencapsulation of natural compounds obtained from essential oil that possess repellent activity. These nanosystems (termed as hosts) provide a hydrophobic environment that accommodate and stabilize small organic molecules (termed as guest) for controlled release and create an external environment that allows solubility in an aqueous phase, thus favoring the preparation of pharmaceutical formulations with cosmetic applications (Figure 3) [32].

Nanoencapsulation has been an alternative and interesting method for the development of a new generation repellent systems against arthropods [33]. The present review exhibits and discusses the main organic matrices used for the nanoencapsulation of natural products obtained from essential oils with repellent activity, including cyclodextrins and polymeric micelles. We also discuss the main molecular details involved in the interaction, thermal stability, and controlled release of these compounds from these organic matrices.

## 2. Natural Compounds with Repellent Activity

Natural products such as essential oils have been widely studied over the last decades due to their cosmetic and pharmaceutical applications. These natural products are extracted from a wide range of botanical materials, and they are characterized by the presence of high diversity of volatile organic compounds [25,26]. Approximately 3000 essential oils were analyzed to date, and only about 10% are commercially available as repellents [34]. The total content, fractions, and isolated constituents of essential oils have been widely reported with larvicide, repellent, and insecticide activities against different species of arthropods that are vectors of human diseases, which makes them especially relevant for the bioprospecting of bioactive molecules with activity against arthropods [35,36]. In addition, due to their natural origin, compounds from essential oils are considered less toxic to the environment as they are more easily biodegradable [34]. Essential oils are rich in lipophilic and volatile compounds, and their encapsulation provides an efficient approach to modulate controlled release, increasing the physical stability and protecting them against environmental degradation [37,38]. These natural products have been interesting candidates as repellents and contribute to the decrease of environmental contamination and better effective control of pests and diseases transmitted by these vectors [39]. Furthermore, these natural compounds obtained from different botanical species show interesting concentrations for isolation that allow the evaluation of their repellent activity [40,41].

The choice of extraction method to obtain a natural product is decisive to determining the type, quantity, and stereochemical structure of the compounds obtained from the essential oils [42]. Among the various methods used to obtain essential oils are microwave-assisted extraction [43] hydrodistillation [44], etc. The use of plants as mosquito repellents has a high level of acceptance in the consumer market due to the perception that natural repellents are safer than synthetic ones [45].

The essential oil from lemon eucalyptus (*Eucalyptus citriodora*) is one of the most effective repellents of natural origin, and its main constituent is citronellal (85%). Other compounds, such as citronellol, limonene, and linalool are also found in this oil. However, pure lemon eucalyptus essential oil is not recommended as an insect repellent by the U.S. Environmental Protection Agency, and there are no studies on its effectiveness and safety [34].

Citronella (*Cymbopogon winterianus*, *Cymbopogon nardus*) essential oil is the most commonly found in insect repellent formulations [46,47]. The commercial products based on citronella contain up to 64% of the natural constituent PMD, which is mainly responsible for the effectiveness and protection against biting insects and other arthropods [15,48,49]. Citronella essential oil is also obtained from the *C. citratus*, and its main constituents include citronellal, citronellol, geraniol, citral, α-pinene, and limonene which are comparable with the mode of action of DEET [50]. This essential oil has been used as a topical insect repellent under the guidance of the United States Environmental Protection Agency despite being toxic and potentially carcinogenic to humans. Some active compounds in essential oils have shown repellent activity and include limonene, 1,8-cineole, geraniol, and citronellal [47].

Limonene is the main constituent of essential oils of lemon (*Citrus limon*) and black pepper (*Piper nigrum*) showing a repellent activity against the genera *Aedes* sp. and *Culex* sp. In addition, the 1,8-cineole, the main component of eucalyptus (*Eucalyptus globulus*) essential oil, showed activity against *A. aegypti* [32]. The compound geraniol is one of the main constituents of essential oils of geranium (*Pelargonium graveolens*) and ginger (*Zingiber officinale* Roscoe), and it has also shown repellent activity against *A. aegypti* and *A. albopictus*. Eugenol, a phenylpropanoid found in different botanical species, such as *Syzygium aromaticum*, *Eugenia caryophyllata*, and *Eugenia aromaticum*, has been widely investigated due to its insect repellent activity. The use of eugenol has been reported as safe, and its carcinogenic potential or other adverse effects need to be further investigated [51,52].

A successful strategy to overcome the limitations related to the high volatility and instability due to external factors (as light, heat, humidity and oxygen) of chemical constituents obtained from the essential oils consists of their nanoencapsulation using polymeric/lipid nanosystems.

Nanoencapsulation offers advantages for the use of volatile and photolabile active compounds from their dispersion or encapsulation in polymeric matrices, adding greater stability, biocompatibility, and efficiency to the potential activity of these natural compounds [47,52,53]. In this context, nanoencapsulation offers protection to the compounds, prolonging their activity through the gradual and controlled release, thus reducing the need for high initial doses or frequent application of the product [54]. In addition, these nanostructures can minimize undesired toxic effects on nontarget organisms, as well as improve physicochemical stability, reducing volatility and preventing degradation of the active compound [55,56]. Currently, several studies analyze different methodologies and techniques aiming at the encapsulation of essential oils and their isolated constituents with repellent activity, obtaining inclusion complexes, microcapsules, nanoemulsions, solid lipid nanoparticles, micelles, liposomes, which are promising alternatives to traditional repellent formulations (Table 1).

**Table 1:** Polymeric/lipid nanosystems applied in the nanoencapsulation of natural compounds obtained from essential oils.

Natural Product			Polymeric/Lipid Nanosystems			References
Essential Oil	Compound Extracted from Essential Oil (Major Compunds)	Technique	Organic Matrices	Structures	Formulations	
-	Eugenol	Co-precipitation/Solvent evaporation	Oligosaccharide	β-CD	Inclusion complex	[51]
Lemongrass (*Cymbopogon flexuosus*)	citral and geraniuml	Co-precipitation	Oligosaccharide	β-CD	Inclusion complex	[39]
Geranium Egyptian (*Pelargonium graveolens*)	β-citronellol					
Lemon Eucalyptus (*Eucalyptus citriadora*)	β-citronellal					
(Rosemary) *Lipia gracilis*	Carvacrol	Kneading, co-evaporation and physical mixture	Oligosaccharide	β-CD	Inclusion complex	[57]
Cedar (*Cedrus atlantica*)		Esterifying	Oligosaccharide/citric acid (citrate)	β-CD/citrate	Inclusion complex	[58]
Lavender (*Lavandula officinalis*)						
Peppermint (*Mentha piperita* L.)						
Cloves (*Eugenia caryophyllus*)	-					
Eucalyptus (*Eucalyptus citriodora*)						
Jasmine (*Jasminum officinale*)						
Citronella (*Cymbopogon winterianus*)	Citronellal Citronellol	Kneading	Oligosaccharide	β-CD	Inclusion complex	[59]
Orange (*Citrus sinensis* L.)	R-limonene	Paste complexation, coprecipitation and physical mixture	Oligosaccharide	β-CD	Inclusion complex	[60]
-	Carvacrol Linalool	Ultrafiltration and centrifugation	Oligosaccharide/chitosan glycol	β-CD/chitosan	Inclusion complex	[61]
Copaiba oilresin (*Copaifera multijuga* Hayne)	β-caryophyllene	Physical mixture, kneading and slurry	Oligosaccharide	β-CD and HPβCD	Inclusion complex	[62]
-	Geraniol	Physical mixture, slurry and paste	Oligosaccharide	β-CD	Inclusion complex	[63]
Rosemary-pepper (*Lippia origanoides*)	Thymol	Freeze-drying/microemulsion	Stearic acid, oleic acid, soybean lecithin and polysorbate 80/HPβCD	NLC/HPβCD	-	[64]
Geranium (*Pelargonium graveolens*)	-	Ultrasonic solvent emulsification	Stearic acid, soybean lecithin and Tween-80	SLN	nanoformulation	[65]
-	Mixture of icaridin (synthetic) and geraniol (natural)	Emulsion/solvent evaporation	Tripalmitin, polyvinyl alcohol) and hydroxypropyl methylcellulose	NLC/SLN	nanoformulation	[66]
Black cumin (*Nigella Sativa L.*)	-	Hot homogenisation	Hydrogenated palm oil, Sorbitol and polysorbate 80	SLN	nanoformulation	[67]
-	Citral	High-pressure homogenization	Glyceryl monostearate, Tween-80 and Span-80	SLN	nanoformulation	[68]
-	D-limonene	Phase transition composition	Polyoxyethylene (20,40, 60 and 80)	-	nanoemulsion	[69]
Eucalyptus oil (*Eucalyptus citriodora*)	-	Uultrasonication	Tween80	-	Nanoemulsion	[70]
Citronella oil (*Cymbopogon winterianus*)	D-Limonene	Cavitation assisted	Tween80 and SPAN80	-	Nanoemulsion	[71]
-	Thymol-eugenol mixtures	Solubilization	Poly (ethylene oxide)/PEO and poly(propylene oxide)/PPO	-	Nanoemulsion	[72]
-	Eugenol	Solubilization	PEO and PPO	-	Polymeric Micelles	[73]
	1,8-Cineole					
	Geraniol					
	Linalool					
	Carvacrol					
	Citronellol					
	Thymol					
	Menthol					
	α-terpineol					
	Nonyl alcohol					
Clove oil (*Eugenia caryophyllus*)	Eugenol	Mixture and Spray dryer	Casein	-	Polymeric Micelles	[74]
Clove oil (*Eugenia caryophyllus*)	Eugenol	Ethanol injection	soybean phospholipid	-	Lipossome	[75]
Thyme essential oil	-	Thin film dispersion	ε-polylysine (Polyvinylpyrrolidone)/Oligosaccharide	β-CD	Lipossome/β-CD	[76]

## 3. Polymerics Systems Applied in the Nanoencapsulation of Natural Products

Demand for safer and eco-friendly repellents has grown in recent years. The vast majority of pharmaceutical and cosmetic formulations applied in the development of repellents against arthropods are based on controlled release systems of compounds that provide prolonged modes of action [77]. The nanosystems used in the development of controlled release formulations include polymers (synthetic and natural) and lipids which have shown low cost, low toxicity, satisfactory biocompatibility, and biodegradability [61].

Repellent formulations based on essential oil without controlled release systems tend to show low efficiency due to the presence of compounds with low molecular weight, high volatility, and instability, which provide short-term repellent protection [58]. Cyclodextrins, polymeric micelles, solid lipid nanoparticles (SLNs), and liposomes are some of the most widely used polymeric systems applied in the nanoencapsulation of essential oils and their isolated natural compounds that possess repellent activity (Figure 3).

### 3.1. Inclusion Complexes Using Cyclodextrins

**Figure 4:**
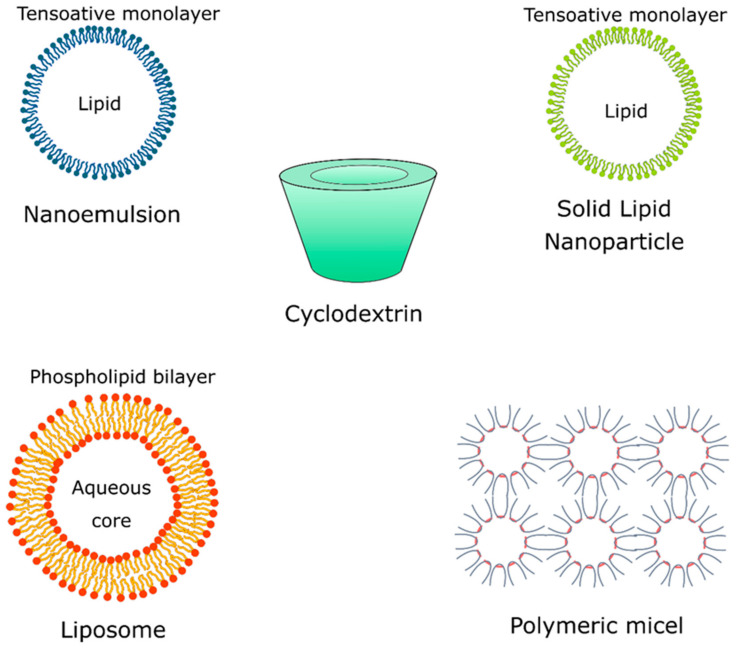
Polymeric/lipid nanosystems applied in the nanoencapsulation of essential oils and their chemical constituents with repellent activity.

**Figure 5:**
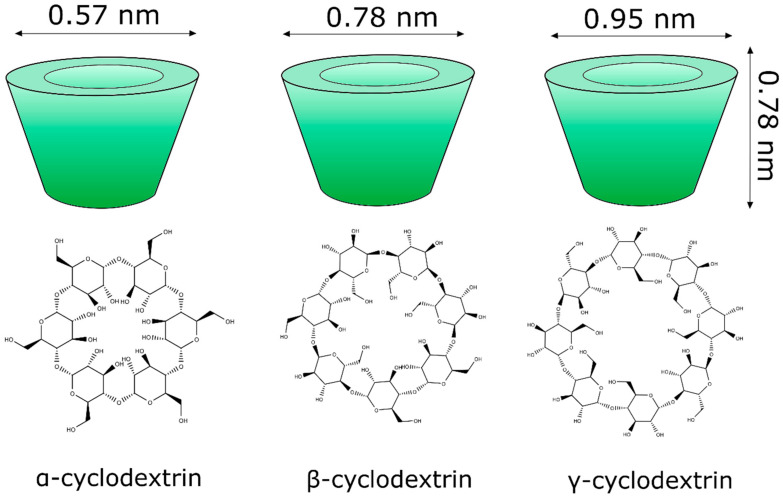
Molecular structure of the main classes of cyclodextrins (α, β, and γ) applied in the nanoencapsulation of natural products.

Cyclodextrins (Figure 4) are cyclic glucose (α-d-glucopyranose) oligosaccharides, produced from enzymatic conversion, degradation, and amide cyclization [47]. Cyclodextrins have a cone-shaped three-dimensional structure with a hydrophilic external surface and hydrophobic cavity. These structures are classified based on the number of d-glucopyranose units, and the most common are cyclodextrins α, β, and γ (Figure 5), which contain six, seven, and eight units, respectively, which are linked to each other by α-1,4 glycoside. Among these organic matrix, beta-cyclodextrin is the most commonly used due to its simple synthesis, availability, cavity diameter, low cost, low irritability to the skin, and absence of mutagenic effects [78].

The internal diameter of the β-cyclodextrin cavity can accommodate aromatic compounds, such as volatile molecules with molecular weights between 200 and 800 g mol^−1^ [79,80].

The physicochemical properties of cyclodextrins (alpha, beta and gamma) are varied. For example, in terms of water solubility, beta-cyclodextrin has the lowest solubility [81].

Cyclodextrins have been used to accommodate a wide range of natural compounds with industrial applications, such as repellents, dyes, insecticides, and herbicides [82,83].

Cyclodextrin reduces the evaporation rate of volatile compounds, thus favoring the controlled release under desired conditions, and these structures also protect the molecules from oxidation and other enzymatic or biological processes, improving the efficiency and time of action of these compounds [63,66,78,84].

Due to the labile structures of natural compounds obtained from essential oils, inclusion complexes containing β-cyclodextrins have been applied to increase the solubility and physicochemical stability of these natural products in water [60,64]. Depending on the properties of the host molecule and the nature of the chosen subclass, the cyclodextrins can be obtained using different methods, such as physical mixing, kneading, atomization, freeze-drying, spray drying, or coprecipitation [78,85,86]. The formation of inclusion complexes between essential oils and cyclodextrins, especially β-cyclodextrins and its derivative HPβCD (hydroxypropyl/β-cyclodextrins), favors the increase of the solubility of lipophilic molecules and protects them against degradation and volatilization [47].

Recently, a study evaluated the formation of inclusion complexes between eugenol and β-cyclodextrin using X-ray diffraction and Fourier transform infrared spectroscopy techniques and revealed the guest/host interaction and, therefore, the success in the complexation process [51]. In another study, essential oils of geranium, lemongrass, and lemon eucalyptus, which have known repellent activity against ticks, were nanoencapsulated by the inclusion process using beta-cyclodextrin as the organic matrix. Inclusion complexes have also been evaluated regarding their larvicidal activity against mosquitoes [39]. Another study showed that the *Citrus sinensis* essential oil present in the β-cyclodextrin inclusion complexes exhibited an interesting larvicidal activity with 100% mortality of larvae of *Aedes aegypti* after 24 h of application [60].

In another recent research, the development of an inclusion complex between β-cyclodextrins and *Lippia gracilis* essential oil using thermal analysis and gas chromatography was studied. The inclusion complexes showed an improved larvicidal activity of the essential oil against the *A. aegypti* when compared with the application of pure essential oil [87]. Furthermore, the complexation of essential oils with β-cyclodextrin has been extensively investigated for pharmaceutical and cosmetic purposes [32].

Khanna and Chakraborty [58] evaluated the inclusion complex formation between β-cyclodextrins and citrate and other essential oils (e.g., cedar, clove, eucalyptus, peppermint, lavender, and jasmine) to assess the repellent effectiveness against the *Anopheles stephensi*, the vector of malaria. The authors identified that lavender (*Lavandula officinalis*) and cedar (*Cedrus atlantica*) oil provided the longest protection times against the mosquito (210 and 160 min, respectively). The citronella release rate from formulations without nanoprotection (normal citronella oil) was higher than that from formulations containing inclusion complexes, indicating that inclusion complexes with beta-clicodextrin could delay the release of citronella oil, increasing its effectiveness. As verified by the authors, citronella nanoformulations with inclusion complexes showed better repellent activity against *A. aegypti* mosquito [59].

Menezes et al. [63] produced a solid-state β-cyclodextrin inclusion complex containing geraniol that was obtained by physical mixing and suspension methods. The structures were analyzed by differential scanning calorimetry, thermogravimetry, Karl Fisher analysis, scanning electron microscopy, and Fourier transforms infrared spectroscopy to verify their formation and structural characteristics. The thermal analysis indicated the formation of complexes by the slurry suspension methods obtained better results for the complexation of geraniol with β-cyclodextrin.

The encapsulation of inclusion complexes in nanostructured systems has been an interesting approach to obtain the benefits of the inclusion complex and lipid nanoparticles such as associations with liposomes and nanostructured lipid carriers [64]. Functionalized cyclodextrins have been widely studied as possible new repellents. Recently studies have demonstrated the synthesis of chitosan nanoparticles functionalized with β-cyclodextrin containing carvacrol and linalool with increased water solubility. A decrease in toxicity was observed when the compounds were nanoencapsulated using the chitosan/beta-cyclodextrin formulation. These nanoparticles presented insecticide activity against the Helicoverpa armigera and Tetranychus urticae species, which can contribute to the effective control of pests [84].

### 3.2. Solid Lipid Nanoparticles (SLNs)

Solid lipid nanoparticles (SLN) (Figure 4) are constituted of lipids and emulsifiers, and these nanostructures show interesting properties for the encapsulation of natural compounds, such as high surface area, high load capacity, and thermal stability, as well as feasibility to incorporate lipophilic and hydrophilic compounds. Lipids also offer better stability and control the compound release and the emulsifiers stabilize the dispersion of lipids [88]. Studies reported that the combination of emulsifiers can avoid particle agglomeration more efficiently. These lipid systems are made of physiological lipids (e.g., mono-or triglyceride mixtures and fatty acids) in mixtures with surfactants and water, which reduces their risks of toxicity, offering biodegradable and biocompatible properties, which in turn make them candidates for repellent formulations [87].

Studies reported that SLN containing geraniol have shown an interesting controlled release [65]. In another study, the essential oil of black cumin (*Nigella sativa*) incorporated into SLNs controlled the evaporation rate of the oil over 48 h at 35 °C during storage. The study showed that SLN systems reduced the volatility and degradation and improved the stability of the compounds, thus maintaining the effective minimum amount to their mode of action [32,65,67]. Research developed by Tian et al. [68], evaluated the thermal and chemical stabilities of SLN containing citrus oil and demonstrated that the thermal stability of the citrus was improved when it was encapsulated using SLN and remained stable during 12 days of storage at 37 °C.

SLN and inclusion complexes of cyclodextrins have been combined in the nanostructured systems [64]. Nanostructured lipid carriers produced with cyclodextrin inclusion complexes and essential oil of *Lippia* sp. successfully combined the advantage of stabilizing the inclusion complexes and controlled release provided by nanoparticles. A second generation of lipid nanoparticles (NLC) has been developed to overcome limitations related to SLN such as limited load capacity and possible expulsion of the active compound during storage. The NLC are characterized by the presence of a liquid lipid along with the solid lipid, which forms an amorphous structure that allows a higher load of active compounds, thus avoiding the loss of the compound during storage [65].

### 3.3. Liposomes

Liposomes (Figure 4) are vesicular structures formed from the hydrophilic aqueous nucleus and a lipophilic phospholipid bilayer. These nanostructures are biocompatible and can accommodate hydrophilic and lipophilic compounds in their compartments, and show as main advantages the reduced evaporation rate, prolonged controlled release and action time, low cutaneous permeation, and low toxicity [33]. The encapsulation of an active compound in liposomes allows protection against degradation and increased solubility. Studies of the essential oil of Santolina (*Santolina insularis*) incorporated into liposomes suggested that these nanostructures show interesting stability and a stable composition over one year and satisfactory protection against degradation when compared with the pure essential oil [89]. A study investigated the stability of clove essential oil and its major component, eugenol, when encapsulated in natural liposomes of soy phospholipids, prepared by the ethanol injection method. The authors demonstrated that the liposomes protected the eugenol from the degradation induced by exposure to ultraviolet rays, without reducing its activity [75]. Lin et al. [76] have developed an interesting system for thyme (*Thymus vulgaris*) essential oil encapsulation using solid liposomes coated with ε-polylysine. These new solid nanosystems showed high stability and longer storage time compared to traditional aqueous liposomes.

### 3.4. Nanoemulsions

Nanoemulsions (Figure 4) are stable isotropic dispersions formed by two immiscible liquids (oil and water) stabilized by surfactants, with mean sizes of 1–50 nm [90,91,92].

These systems are usually represented by a pseudo-ternary phase diagram, where their sides represent a binary mixture of surfactant-co-surfactant, organic molecule-water, and organic molecule (e.g., herbicide, repellent, and drug). These nanostructures are formed only in a specific and narrow range of concentrations for a given surfactant-oil-water structure [93].

Thus, nanoemulsions can be divided into: (1) oil in water, in which the oil phase is dispersed in a continuous aqueous phase; (2) water in oil, where the aqueous phase is dispersed in a continuous oil phase, and (3) bicontinuous emulsions in which the micro-domains of the oil and water phases are interspersed [92]. Different formulations of essential oils in nanoemulsions have shown antimicrobial, antiviral, analgesic, anti-inflammatory, antioxidant and antibacterial activities and can effectively mitigate insect bites when applied topically [94]. These nanoemulsions have also been applied to protect the bioactive compounds from essential oils of environmental degradation [95].

Repellent formulations containing nanoemulsions are promising to reduce mosquito-borne diseases and to minimize the limitations related to irritation and dryness of the skin. These properties are due to the intrinsic physicochemical properties of nanoemulsions, such as uniformity and reduced sizes of nanostructures (20–200 nm), low viscosity, and interesting optical transparency [96,97]. In the last few years, interest in research on nanoemulsified repellents has increased due to their pharmaceutical and cosmetic applications [98].

These systems show several improved properties when compared with common emulsions that provide their applications in repellent formulations. These properties include better physical stability and droplet aggregation, efficient permeation, improved bioavailability, better water solubility, adjustable loading capacity, better chemical stability, slow release of the bioactive compounds, low cost, and lower toxicity [69,72,95].

A study reported a satisfactory performance of the nanoemulsion containing the eucalyptus essential oil (*Eucalyptus globulus*) against the mosquito *Culex quinquefasciatus*, where the activity of the nanoemulsions was related to the size of the oil droplets [70]. Similarly, another study showed the effect of citronella oil nanoemulsions against the *A. aegypti* mosquito [71]. Recently, Lucia et al. [72] evaluated the stability of essential oils based on nanoemulsions after 28 months, and the authors noticed that samples containing eugenol oil did not show a significant change in their homogeneity after 28 months, maintaining their monodisperse properties and constant droplet size.

### 3.5. Polymeric Micelles

Polymeric micelles (Figure 4) are spherical colloidal particles with a hydrophobic core and a hydrophilic outer layer that can be used as mosquito repellent systems, as well as controlled release systems, due to their high encapsulation capacity. These nanostructures are characterized as block copolymer structures mounted on a core containing the active substance. Encapsulation of the active compound can be produced by chemical conjugation or physical entrapment [99]. Polymeric micelles can be prepared from polymers and copolymers, among them we can cite polyethylene glycol (PEG), polycaprolactone (PCL), polylactic acid (PLA), poly(histidine), poly(aspartic acid), poly(isopropyl acrylamide)), poly-(2-ethyl-2-oxazoline), poly (2-dimethylaminoethyl), poly (ethyleneimine), poly (dimethylamine methacrylate), and poly-(ethylene oxide) [32,73]. These nanostructures have a wide range of applications as insect repellents against different species.

Barradas et al. [100] reported that the DEET added in micellar formulations based on a triblock copolymer formed by poly (ethylene oxide)-poly (propylene oxide)-poly(ethylene oxide) exhibited slow release for more than seven hours. Other studies have demonstrated that essential oils, consisting of monoterpenes, such as linalool, 1,8-cineol, α-terpineol, thymol, eugenol, and geraniol when encapsulated in polymeric nanomicelles by the process of solubilization by homogenization showed interesting repellent activity to control *Pediculus humanus capitits* (lice). The micellar formulation was effective showing mortality above 60% against the insect species. The most effective system contained in their composition linalool, 1,8-cineole, terpineol, thymol, eugenol, and geraniol [73].

Putri et al. [74] microencapsulated the eugenol, present in clove essential oil (*Syzygium aromaticum*), using the casein micelle technique. The percentage of encapsulated eugenol was 87.99%, and the tests were performed against the *Apis mellifera*, 24 h after encapsulation. The encapsulated eugenol proved to be efficient at killing bees and has a lower toxicity value when compared to pure eugenol and a commercial insecticide (DTX multi-insecticide—pralethrin and cypermethine).

## 4. Molecular Details of Inclusion Complexes Formed between Natural Compounds from Essential and Organic Matrices

Several computational analyses have been performed as complementary methods to the in vivo and in vitro repellent assays aiming to investigate the molecular mechanism of action of repellents and to improve their activity [21,101]. These computational approaches include molecular docking, density functional theory (DFT), molecular dynamics simulations, and binding free energy calculations. These methods can evaluate the physicochemical properties, including lipophilicity, shape, stereochemistry, electrostatic surface, and intermolecular interactions formed with the repellents and organic matrices. They can also evaluate possible conformational changes induced by the repellent binding with the molecular target involved with olfactory recognition [101,102,103].

Recently, a study used QSAR models to evaluate the larvicidal activity of 50 constituents of essential oils against the *Culex quinquefasciatus* to identify the molecular and structural properties for the larvicidal activity. In this study, molecular docking of α-humulene and β-caryophyllene on sterol carrier protein-2 (SCP-2) was obtained from the database of the National Center for Biotechnology Information (NCBI) and used to investigate the molecular mechanism of action of these compounds. In this work, the QSAR models showed that the structural property of π bonds is what most contributes to larvicidal activity. Docking studies, on the other hand, showed a good interaction capacity of the SCP-2 protein with the molecules tested, with α-humulene and β-caryophyllene being the compounds with the highest binding energy [104].

Cyclodextrins and their inclusion complexes have been studied using different computational methods, such as molecular dynamics and Monte Carlo simulations [105,106,107]. Alvira [102] performed a theoretical study aiming to evaluate the formation of the inclusion complex between the β-cyclodextrin (β-CD) and eugenol in water using molecular dynamics simulations. In this study, the van der Waals terms better contributed with the total energy, which directly determined the conformational configuration of the inclusion complex. Regarding the molecular dynamics, the inclusion complex conformations were deduced from the position probability density that represents the preferred location and orientation in the simulation. Two types of configurations were proposed for the inclusion complexes, each one with the hydroxyl and methoxyl groups pointing to a different border of β-CD. The model presented in this study reproduced the ability of eugenol to form inclusion complexes with β-CD with 1:2 stoichiometry, which demonstrated to be more stable than that with 1:1 stoichiometry. These results agree with experimental spectroscopic data. The authors also demonstrated that nonbonded Van der Waals interactions were the main intermolecular forces involved with the stabilization between the host and the guest molecules in both stoichiometries.

Other studies have focused on the analyses of alternative organic matrices for nanoencapsulation of natural compounds. Recently, a study performed the computational analyses of the inclusion complexes formed between the cucurbit[7]uril and dillapiole and its derivative compounds using molecular docking simulation and DFT calculation at the B3LYP-631g(d) level of theory. The authors analyzed the steric constraint associated with the substituent positioning of the dillapiole derivatives and the formation of intermolecular interactions involved with the stabilization of the inclusion complex. The hydrogen bonding, electrostatic, dispersion, and pi-alkyl were the main intermolecular interactions formed in the inclusion complex. Furthermore, the study demonstrated that dilapiolle derivatives with attached H and/or methoxy groups at the benzene ring with primary alcohol exhibited less steric constraint than the derivatives that had two methoxy groups attached to the benzene ring with secondary alcohol [103].

## 5. Final Considerations: Perspectives in the Development of New Nanosystem Repellents

Essential oils have enormous potential to be applied in repellent formulations due to their biological activities such as antioxidants, anti-inflammatory, insecticide, repellent, among others. However, due to its instability, volatile properties, and the presence of some environmental conditions such as light, oxygen, and heat, their effectiveness is impaired or lost before reaching their target locations. Thus, the development of formulations to protect these active compounds is highly demanded by the cosmetic and pharmaceutical industries to increase their effectiveness and promote their controlled release. These formulations can be obtained by nanoencapsulation using polymeric/lipid nanosystems, such as cyclodextrins, polymeric micelles, nanoemulsions, and liposomes. According to the specialized literature, all strategies have shown an interesting ability to improve the stability and effectiveness of the repellent activity of the essential oils, thus increasing their bioavailability in relation to the compounds applied alone. As discussed previously, the selection of the type of formulation and the method of preparation of the carrier nanosystems represent key parameters for obtaining a final formulation with the most appropriate properties for the desired pharmaceutical application, in this case, polymeric/lipid nanosystems based on essential oils, with repellent activity.

Among the polymer systems exhibited in the present review, the cyclodextrins are the most used for the nanoencapsulation of natural compounds with repellent activity, as they are low cost and effective at protecting active compounds from degradation, improving the repellent activity of these compounds against the main arthropods that are vectors of diseases.

A direct comparison of the different controlled-release nanosystems is not easily achievable due to the substantial differences between them in terms of structure and applications. In particular, in designing the suitable nanosystem for the controlled release of a specific natural compound includes different variables such as the method of synthesis, the selection of biocompatible raw materials, and the structure of the nanocarrier must be taken into account. In this context, theoretical computational studies, based on molecular mechanics and molecular dynamics have shown great relevance, because they allow us to predict the main molecular interactions between polymeric/lipid nanosystems and natural compounds, the thermal stability and the solubility of the host-guest complexes, and the main molecular interactions of these isolated natural products with the olfactory systems of mosquitoes which are the main molecular targets of the repellent compounds.

The research for new mosquito repellents is increasing due to the recurrence of various diseases transmitted by mosquito bites worldwide, so natural products represent an interesting solution for the development of new repellent formulations with less environmental impact. Future research should be conducted for the use of nanoencapsulation in the development of controlled release systems containing essential oils with repellent activity. In addition, these researches should be aimed at developing patentable products using cost-effective methods that are also applicable to the industrial scale of pharmaceutical and cosmetic companies. Therefore, the future development of in vivo studies of repellent activity must be based on the results obtained from computational analyses of the host-guest nanosystems to provide reliable results of pharmaceutical interest, thus allowing the pharmaceutical application of the newest controlled release systems of the repellent compounds.

## References

[1] Tabanca N., Bernier U.R., Agramonte N.M., Tsikolia M., Bloomquist J.R. (2016). Discovery of Repellents from Natural Products. Curr. Org. Chem..

[2] Norris E.J., Coats J.R. (2017). Current and Future Repellent Technologies: The Potential of Spatial Repellents and Their Place in Mosquito-Borne Disease Control. Int. J. Environ. Res. Public Health.

[3] Ha T.S., Smith D.P. (2009). Odorant and pheromone receptors in insects. Front. Cell. Neurosci..

[4] Zheng W., Peng W., Zhu C., Zhang Q., Saccone G., Zhang H. (2013). Identification and expression profile analysis of odorant binding proteins in the oriental fruit fly Bactrocera dorsalis. Int. J. Mol. Sci..

[5] Boyle S.M., McInally S., Ray A. (2013). Expanding the olfactory code by in silico decoding of odor-receptor chemical space. eLife.

[6] Zhang Y., Ren Y., Wang X., Liu Y., Wang N. (2019). Responses to Host Plant Volatiles and Identification of Odorant Binding Protein and Chemosensory Protein Genes in Bradysia odoriphaga. ACS Omega.

[7] Venthur H., Zhou J.-J.J. (2018). Odorant receptors and odorant-binding proteins as insect pest control targets: A comparative analysis. Front. Physiol..

[8] Damberger F., Nikonova L., Horst R., Peng G., Leal W.S., Wüthrich K. (2000). NMR Characterization of a pH-Dependent Equilibrium between Two Folded Solution Conformations of the Pheromone-Binding Protein from Bombyx Mori. Protein Sci..

[9] Manoharan M., Fuchs P.F.J., Sowdhamini R., Offmann B. (2014). Insights on pH-dependent conformational changes of mosquito odorant binding proteins by molecular dynamics simulations. J. Biomol. Struct. Dyn..

[10] Jin X., Ha T.S., Smith D.P. (2008). SNMP is a signaling component required for pheromone sensitivity in Drosophila. Proc. Natl. Acad. Sci. USA.

[11] Meepagala K.M., Bernier U.R., Burandt C., Duke S.O. (2013). Mosquito repellents based on a natural chromene analogue with longer duration of action than N,N-diethyl-meta-toluamide (DEET). J. Agric. Food Chem..

[12] Sfara V., Mougabure-Cueto G., Zerba E.N., Alzogaray R.A. (2011). Adaptation of the repellency response to DEET in Rhodnius prolixus. J. Insect Physiol..

[13] Alzogaray R.A., Fontan A., Zerba E.N. (2000). Repellency of deet to nymphs of Triatoma infestans. Med. Vet. Entomol..

[14] Syed Z., Leal W.S. (2008). Mosquitoes smell and avoid the insect repellent DEET. Proc. Natl. Acad. Sci. USA.

[15] Diaz J.H. (2016). Chemical and plant-based insect repellents: Efficacy, safety, and toxicity. Wilderness Environ. Med..

[16] Iliou K., Kikionis S., Petrakis P.V., Ioannou E., Roussis V. (2019). Citronella oil-loaded electrospun micro/nanofibrous matrices as sustained repellency systems for the Asian tiger mosquito Aedes albopictus. Pest. Manag. Sci..

[17] Stanczyk N.M., Brookfield J.F.Y., Field L.M., Logan J.G. (2013). Aedes aegypti Mosquitoes Exhibit Decreased Repellency by DEET following Previous Exposure. PLoS ONE.

[18] Stanczyk N.M., Brookfield J.F.Y., Ignell R., Logan J.G., Field L.M. (2010). Behavioral insensitivity to DEET in Aedes aegypti is a genetically determined trait residing in changes in sensillum function. Proc. Natl. Acad. Sci. USA.

[19] Thireou T., Kythreoti G., Tsitsanou K.E., Koussis K., Drakou C.E., Kinnersley J., Kröber T., Guerin P.M., Zhou J.J., Iatrou K. (2018). Identification of novel bioinspired synthetic mosquito repellents by combined ligand-based screening and OBP-structure-based molecular docking. Insect Biochem. Mol. Biol..

[20] González-González A., Palma-Millanao R., Yáñez O., Rojas M., Mutis A., Venthur H., Quiroz A., Ramírez C.C. (2016). Virtual screening of plant volatile compounds reveals a high affinity of Hylamorpha elegans (Coleoptera: Scarabaeidae) odorant-binding proteins for sesquiterpenes from its native host. J. Insect Sci..

[21] Da Costa K.S., Galúcio J.M., Da Costa C.H.S., Santana A.R., Dos Santos Carvalho V., Do Nascimento L.D., Lima E Lima A.H., Neves Cruz J., Alves C.N., Lameira J. (2019). Exploring the Potentiality of Natural Products from Essential Oils as Inhibitors of Odorant-Binding Proteins: A Structure- and Ligand-Based Virtual Screening Approach to Find Novel Mosquito Repellents. ACS Omega.

[22] Murphy E.J., Booth J.C., Davrazou F., Port A.M., Jones D.N.M. (2013). Interactions of anopheles gambiae odorant-binding proteins with a human-derived repellent: Implications for the mode of action of N,N-diethyl-3-methylbenzamide (DEET). J. Biol. Chem..

[23] Chauhan K.R., Klun J.A., Debboun M., Kramer M. (2005). Feeding deterrent effects of catnip oil components compared with two synthetic amides against Aedes aegypti. J. Med. Entomol..

[24] Yadav N.P., Rai V.K., Mishra N., Sinha P., Bawankule D.U., Pal A., Tripathi A.K., Chanotiya C.S. (2014). A novel approach for development and characterization of effective mosquito repellent cream formulation containing citronella oil. Biomed. Res. Int..

[25] Do Nascimento L.D., de Moraes A.A.B., da Costa K.S., Galúcio J.M.P., Taube P.S., Costa C.M.L., Cruz J.N., de Aguiar Andrade E.H., de Faria L.J.G. (2020). Bioactive natural compounds and antioxidant activity of essential oils from spice plants: New findings and potential applications. Biomolecules.

[26] Nerio L.S., Olivero-Verbel J., Stashenko E. (2010). Repellent activity of essential oils: A review. Bioresour. Technol..

[27] Shah S.I., Khutoryanskiy V.V., Williams A.C. (2021). A novel polymer insect repellent conjugate for extended release and decreased skin permeation of para-menthane-3,8-diol. Pharmaceutics.

[28] Wu W., Li S., Yang M., Lin Y., Zheng K., Akutse K.S. (2020). Citronellal perception and transmission by Anopheles gambiae s.s. (Diptera: Culicidae) females. Sci. Rep..

[29] Müller G.C., Junnila A., Butler J., Kravchenko V.D., Revay E.E., Weiss R.W., Schlein Y. (2009). Efficacy of the Botanical Repellents Geraniol, Linalool, and Citronella against Mosquitoes. J. Vector Ecol..

[30] Ditzen M., Pellegrino M., Vosshall L.B. (2008). Insect Odorant Receptors Are Molecular Targets of the Insect Repellent DEET. Science.

[31] Dogan E.B., Ayres J.W., Rossignol P.A. (1999). Behavioural mode of action of deet: Inhibition of lactic acid attraction. Med. Vet. Entomol..

[32] Mapossa A.B., Focke W.W., Tewo R.K., Androsch R., Kruger T. (2021). Mosquito-repellent controlled-release formulations for fighting infectious diseases. Malar. J..

[33] Tavares M., da Silva M.R.M., de Oliveira de Siqueira L.B., Rodrigues R.A.S., Bodjolle-d’Almeira L., dos Santos E.P., Ricci-Júnior E. (2018). Trends in insect repellent formulations: A review. Int. J. Pharm..

[34] Lee M.Y. (2018). Essential Oils as Repellents against Arthropods. Biomed. Res. Int..

[35] Gillij Y.G., Gleiser R.M., Zygadlo J.A. (2008). Mosquito repellent activity of essential oils of aromatic plants growing in Argentina. Bioresour. Technol..

[36] Liang J.Y., Guo S.S., Zhang W.J., Geng Z.F., Deng Z.W., Du S.S., Zhang J. (2018). Fumigant and repellent activities of essential oil extracted from Artemisia dubia and its main compounds against two stored product pests. Nat. Prod. Res..

[37] Do Nascimento L.D., Cascaes M.M., da Costa K.S., de Andrade E.H.A., Andrade E.L., Costa C.M.L., de Faria L.J.G. (2019). Microencapsulamento De Óleos Essenciais: Conceitos e Aplicações. Produção Conhecimento Eng. Química.

[38] Duarte J.L., Taira T.C., Di Filippo L.D., Fonseca-Santos B., Pinto M.C., Chorilli M. (2020). Novel bioadhesive polycarbophil-based liquid crystal systems containing Melaleuca alternifolia oil as potential repellents against Aedes aegypti. J. Mol. Liq..

[39] Hogenbom J., Jones A., Wang H.V., Pickett L.J., Faraone N. (2021). Synthesis and characterization of β-cyclodextrin-essential oil inclusion complexes for tick repellent development. Polymers.

[40] Mathew N., Lalthazuali (2017). Mosquito repellent activity of volatile oils from selected aromatic plants. Parasitol. Res..

[41] Azeem M., Zaman T., Tahir M., Haris A., Iqbal Z., Binyameen M., Nazir A., Shad S.A., Majeed S., Mozūraitis R. (2019). Chemical composition and repellent activity of native plants essential oils against dengue mosquito, Aedes aegypti. Ind. Crops Prod..

[42] Detsi A., Kavetsou E., Kostopoulou I., Pitterou I., Pontillo A.R.N., Tzani A., Christodoulou P., Siliachli A., Zoumpoulakis P. (2020). Nanosystems for the Encapsulation of Natural Products: The Case of Chitosan Biopolymer as a Matrix. Pharmaceutics.

[43] Do Nascimento L.D., Almeida L.Q., de Sousa E.M.P., Costa C.M.L., da Costa K.S., de Andrade E.H.A., de Faria L.J.G. (2020). Microwave-assisted extraction: An alternative to extract Piper aduncum essential oil. Braz. J. Dev..

[44] Diniz Do Nascimento L., Gomes Silva S., Cascaes M.M., Santana Da Costa K., Luis P., Figueiredo B., Leal Costa M., Helena De Aguiar Andrade E., Guerreiro De Faria L.J. (2021). molecules Drying Effects on Chemical Composition and Antioxidant Activity of Lippia thymoides Essential Oil, a Natural Source of Thymol. Molecules.

[45] Tisgratog R., Sanguanpong U., Grieco J.P., Ngoen-Kluan R., Chareonviriyaphap T. (2016). Plants traditionally used as mosquito repellents and the implication for their use in vector control. Acta Trop..

[46] Francikowski J., Baran B., Cup M., Janiec J., Krzyżowski M. (2019). Commercially Available Essential Oil Formulas as Repellents against the Stored-Product Pest Alphitobius diaperinus. Insects.

[47] da Silva M.R.M., Ricci-Júnior E. (2020). An approach to natural insect repellent formulations: From basic research to technological development. Acta Trop..

[48] Prabakaran P., Sivasubramanian C., Veeramani R., Prabhu S. (2017). Review Study on Larvicidal and Mosquito Repellent Activity of Volatile Oils Isolated from Medicinal Plants. Int. J. Environ. Agric. Biotechnol..

[49] Ríos N., Stashenko E.E., Duque J.E. (2017). Evaluation of the insecticidal activity of essential oils and their mixtures against Aedes aegypti (Diptera: Culicidae). Rev. Bras. Entomol..

[50] Maia M.F., Moore S.J. (2011). Plant-based insect repellents: A review of their efficacy, development and testing PMD from lemon eucalyptus (Corymbia citriodora) extract. Malar. J..

[51] De Freitas C.A.B., de Araújo R.C.S., da Paz S.P.A., de Silva J.R.A., Alves C.N., Lameira J. (2021). Obtenção E Caracterização De Complexo De Inclusão De Β-Ciclodextrina E Eugenol/Preparation and Characterization of Β-Cyclodextrin Inclusion Complex of Eugenol. Braz. J. Dev..

[52] Chiriac A.P., Rusu A.G., Nita L.E., Chiriac V.M., Neamtu I., Sandu A. (2021). Polymeric carriers designed for encapsulation of essential oils with biological activity. Pharmaceutics.

[53] Cimino C., Maurel O.M., Musumeci T., Bonaccorso A., Drago F., Souto E.M.B., Pignatello R., Carbone C. (2021). Essential oils: Pharmaceutical applications and encapsulation strategies into lipid-based delivery systems. Pharmaceutics.

[54] Peres M.C., de Souza Costa G.C., dos Reis L.E.L., da Silva L.D., Peixoto M.F., Alves C.C.F., Forim M.R., Quintela E.D., Araújo W.L., de Melo Cazal C. (2020). In natura and nanoencapsulated essential oils from Xylopia aromatica reduce oviposition of Bemisia tabaci in Phaseolus vulgaris. J. Pest Sci..

[55] Pavela R. (2015). Essential oils for the development of eco-friendly mosquito larvicides: A review. Ind. Crops Prod..

[56] Mossa A.T.H. (2016). Green Pesticides: Essential oils as biopesticides in insect-pest management. J. Environ. Sci. Technol..

[57] Galvão J.G., Cerpe P., Santos D.A., Gonsalves J.K., Santos A.J., Nunes R.K., Lira A.A., Alves P.B., La Corte R., Blank A.F. (2018). Lippia gracilis essential oil in í µí¼·-cyclodextrin inclusion complexes: An environmentally safe formulation to control Aedes aegypti larvae. Pest Manag. Sci..

[58] Khanna S., Chakraborty J.N. (2018). Mosquito repellent activity of cotton functionalized with inclusion complexes of β-cyclodextrin citrate and essential oils. Fash. Text..

[59] Songkro S., Hayook N., Jaisawang J., Maneenuan D., Chuchome T., Kaewnopparat N. (2012). Investigation of inclusion complexes of citronella oil, citronellal and citronellol with b-cyclodextrin for mosquito repellent. J. Incl. Phenom. Macrocycl. Chem..

[60] Galvão J.G., Silva V.F., Ferreira S.G., França F.R.M., Santos D.A., Freitas L.S., Alves P.B., Araújo A.A.S., Cavalcanti S.C.H., Nunes R.S. (2015). β-cyclodextrin inclusion complexes containing Citrus sinensis (L.) Osbeck essential oil: An alternative to control Aedes aegypti larvae. Thermochim. Acta.

[61] Campos E.V.R., Proença P.L.F., Oliveira J.L., Melville C.C., Vechia J.F.D., De Andrade D.J., Fraceto L.F. (2018). Chitosan nanoparticles functionalized with β-cyclodextrin: A promising carrier for botanical pesticides. Sci. Rep..

[62] Gabriel de Oliveira Pinheiro J., de Aragão Tavares E., Santos da Silva S., Félix Silva J., Maria Barbosa Gomes de Carvalho Y., Rhayanny Assunção Ferreira M., Antunes de Souza Araújo A., Guimarães Barbosa E., de Freitas Fernandes Pedrosa M., Alberto Lira Soares L. (2017). Inclusion Complexes of Copaiba (Copaifera multijuga Hayne) Oleoresin and Cyclodextrins: Physicochemical Characterization and Anti-Inflammatory Activity. Int. J. Mol. Sci. Artic..

[63] Menezes P.P., Serafini M.R., Santana B.V., Nunes R.S., Quintans L.J., Silva G.F., Medeiros I.A., Marchioro M., Fraga B.P., Santos M.R.V. (2012). Solid-state β-cyclodextrin complexes containing geraniol. Thermochim. Acta.

[64] Pires F.Q., da Silva J.K.R., Sa-Barreto L.L., Gratieri T., Gelfuso G.M., Cunha-Filho M. (2019). Lipid nanoparticles as carriers of cyclodextrin inclusion complexes: A promising approach for cutaneous delivery of a volatile essential oil. Colloids Surf. B Biointerfaces.

[65] Adel M.M., Salem N.Y., Abdel-Aziz N.F., Ibrahim S.S. (2019). Application of new nano pesticide geranium oil loaded-solid lipid nanoparticles for control the black cutworm agrotis ipsilon (Hub.) (lepi., noctuidae). EurAsian J. Biosci..

[66] Abrantes D.C., Rogerio C.B., de Oliveira J.L., Campos E.V.R., de Araújo D.R., Pampana L.C., Duarte M.J., Valadares G.F., Fraceto L.F. (2021). Development of a Mosquito Repellent Formulation Based on Nanostructured Lipid Carriers. Front. Pharmacol..

[67] Alhaj N.A., Shamsudin M.N., Alipiah N.M., Zamri H.F., Bustamam A., Ibrahim S., Abdullah R. (2010). Characterization of Nigella sativa L. essential oil-loaded solid lipid nanoparticles. Am. J. Pharmacol. Toxicol..

[68] Tian H., Lu Z., Li D., Hu J. (2018). Preparation and characterization of citral-loaded solid lipid nanoparticles. Food Chem..

[69] Feng J., Wang R., Chen Z., Zhang S., Yuan S., Cao H., Jafari S.M., Yang W. (2020). Formulation optimization of D-limonene-loaded nanoemulsions as a natural and efficient biopesticide. Colloids Surf. A Physicochem. Eng. Asp..

[70] Sugumar S., Ghosh V., Nirmala M.J., Mukherjee A., Chandrasekaran N. (2014). Ultrasonic emulsification of eucalyptus oil nanoemulsion: Antibacterial activity against Staphylococcus aureus and wound healing activity in Wistar rats. Ultrason. Sonochem..

[71] Agrawal N., Maddikeri G.L., Pandit A.B. (2017). Sustained release formulations of citronella oil nanoemulsion using cavitational techniques. Ultrason. Sonochem..

[72] Lucia A., Toloza A.C., Fanucce M., Fernández-Peña L., Ortega F., Rubio R.G., Coviella C., Guzmán E. (2020). Nanoemulsions based on thymol-eugenol mixtures: Characterization, stability and larvicidal activity against aedes aegypti. Bull. Insectol..

[73] Lucia A., Toloza A.C., Guzmán E., Ortega F., Rubio R.G. (2017). Novel polymeric micelles for insect pest control: Encapsulation of essential oil monoterpenes inside a triblock copolymer shell for head lice control. PeerJ.

[74] Putri Y.R.P., Pratami D.K., Hermansyah H., Wijanarko A., Sahlan M. (2019). Study controlled release, toxicity test, and pesticide test of microcapsule eugenol with casein micelle. AIP Conf. Proc..

[75] Sebaaly C., Jraij A., Fessi H., Charcosset C., Greige-Gerges H. (2015). Preparation and characterization of clove essential oil-loaded liposomes. Food Chem..

[76] Lin L., Zhu Y., Thangaraj B., Abdel-Samie M.A.S., Cui H. (2018). Improving the stability of thyme essential oil solid liposome by using β-cyclodextrin as a cryoprotectant. Carbohydr. Polym..

[77] Prakash B., Kujur A., Yadav A., Kumar A., Singh P.P., Dubey N.K. (2018). Nanoencapsulation: An efficient technology to boost the antimicrobial potential of plant essential oils in food system. Food Control.

[78] Bezerra F.M., Lis M.J., Firmino H.B., Da Silva J.G.D., Valle R.D.C.S.C., Valle J.A.B., Scacchetti F.A.P., Tessaro A.L. (2020). The role of β-cyclodextrin in the textile industry-review. Molecules.

[79] Kotronia M., Kavetsou E., Loupassaki S., Kikionis S., Vouyiouka S., Detsi A., Chinga Carrasco G. (2017). Encapsulation of Oregano (*Origanum onites* L.) Essential Oil in β-Cyclodextrin (β-CD): Synthesis and Characterization of the Inclusion Complexes. Bioengineering.

[80] Lis M.J., Carmona Ó.G., Carmona C.G., Bezerra F.M. (2018). Inclusion complexes of citronella oil with β-cyclodextrin for controlled release in biofunctional textiles. Polymers.

[81] Sherje A.P., Dravyakar B.R., Kadam D., Jadhav M. (2017). Cyclodextrin-based nanosponges: A critical review. Carbohydr. Polym..

[82] Nardello-Rataj V., Leclercq L. (2014). Encapsulation of biocides by cyclodextrins: Toward synergistic effects against pathogens. Beilstein J. Org. Chem..

[83] Radu C.D., Parteni O., Ochiuz L. (2016). Applications of cyclodextrins in medical textiles—Review. J. Control. Release.

[84] Campos E.V.R., Proença P.L.F., Oliveira J.L., Pereira A.E.S., De Morais Ribeiro L.N., Fernandes F.O., Gonçalves K.C., Polanczyk R.A., Pasquoto-Stigliani T., Lima R. (2018). Carvacrol and linalool co-loaded in β-cyclodextrin-grafted chitosan nanoparticles as sustainable biopesticide aiming pest control. Sci. Rep..

[85] Rakmai J., Cheirsilp B., Mejuto J.C., Torrado-Agrasar A., Simal-Gándara J. (2017). Physico-chemical characterization and evaluation of bio-efficacies of black pepper essential oil encapsulated in hydroxypropyl-beta-cyclodextrin. Food Hydrocoll..

[86] Rakmai J., Cheirsilp B. (2016). Continuous production of β-cyclodextrin by cyclodextrin glycosyltransferase immobilized in mixed gel beads: Comparative study in continuous stirred tank reactor and packed bed reactor. Biochem. Eng. J..

[87] Kelidari H.R., Moemenbellah-Fard M.D., Morteza-Semnani K., Amoozegar F., Shahriari-Namadi M., Saeedi M., Osanloo M. (2020). Solid-lipid nanoparticles (SLN)s containing Zataria multiflora essential oil with no-cytotoxicity and potent repellent activity against Anopheles stephensi. J. Parasit. Dis..

[88] Mehnert W., Mäder K. (2012). Solid lipid nanoparticles: Production, characterization and applications. Adv. Drug Deliv. Rev..

[89] Valenti D., De Logu A., Loy G., Sinico C., Bonsignore L., Cottiglia F., Garau D., Fadda A.M. (2001). Liposome-incorporated *Santolina insularis* essential oil: Preparation, characterization and in vitro antiviral activity. J. Liposome Res..

[90] Pinto I.C., Cerqueira-Coutinho C.S., Santos E.P., Carmo F.A., Ricci-Junior E. (2017). Development and characterization of repellent formulations based on nanostructured hydrogels. Drug Dev. Ind. Pharm..

[91] Nirmala M.J., Nagarajan R. (2017). Recent Research Trends in Fabrication and Applications of Plant Essential Oil Based Nanoemulsions. J. Nanomed. Nanotechnol..

[92] Rai V.K., Mishra N., Yadav K.S., Yadav N.P. (2018). Nanoemulsion as pharmaceutical carrier for dermal and transdermal drug delivery: Formulation development, stability issues, basic considerations and applications. J. Control. Release.

[93] Montenegro L., Lai F., Offerta A., Sarpietro M.G., Micicchè L., Maccioni A.M., Valenti D., Fadda A.M. (2016). From nanoemulsions to nanostructured lipid carriers: A relevant development in dermal delivery of drugs and cosmetics. J. Drug Deliv. Sci. Technol..

[94] Franklyne J.S., Mukherjee A., Chandrasekaran N. (2016). Essential oil micro-and nanoemulsions: Promising roles in antimicrobial therapy targeting human pathogens. Lett. Appl. Microbiol..

[95] Pavoni L., Pavela R., Cespi M., Bonacucina G., Maggi F., Zeni V., Canale A., Lucchi A., Bruschi F., Benelli G. (2019). Green micro-and nanoemulsions for managing parasites, vectors and pests. Nanomaterials.

[96] Bajerski L., Michels L.R., Colomé L.M., Bender E.A., Freddo R.J., Bruxel F., Haas S.E. (2016). The use of Brazilian vegetable oils in nanoemulsions: An update on preparation and biological applications. Braz. J. Pharm. Sci..

[97] Zengin G., Chen L., Yaouba S., Narawi M.M., Zain M.N., Mohd Narawi M., Ing Chiu H., Keong Yong Y., Nadhirah Mohamad Zain N., Raoov Ramachandran M. (2020). Biocompatible Nutmeg Oil-Loaded Nanoemulsion as Phyto-Repellent. Front. Pharmacol..

[98] Echeverría J., Albuquerque R. (2019). Nanoemulsions of Essential Oils: New Tool for Control of Vector-Borne Diseases and In Vitro Effects on Some Parasitic Agents. Medicines.

[99] Balaji A.P.B., Mishra P., Suresh Kumar R.S., Mukherjee A., Chandrasekaran N. (2015). Nanoformulation of poly(ethylene glycol) polymerized organic insect repellent by PIT emulsification method and its application for Japanese encephalitis vector control. Colloids Surf. B Biointerfaces.

[100] Barradas T.N., Lopes L.M.A., Ricci E., De Holanda E Silva K.G., Mansur C.R.E. (2013). Development and characterization of micellar systems for application as insect repellents. Int. J. Pharm..

[101] Dickens J.C., Bohbot J.D. (2013). Mini review: Mode of action of mosquito repellents. Pestic. Biochem. Physiol..

[102] Alvira E. (2018). Theoretical study of the β-cyclodextrin inclusion complex formation of eugenol in water. Molecules.

[103] Mustafa S.F.Z., Arsad S.R., Mohamad H., Abdallah H.H., Maarof H. (2021). Host-guest molecular encapsulation of cucurbit[7]uril with dillapiole congeners using docking simulation and density functional theory approaches. Struct. Chem..

[104] Andrade-Ochoa S., Correa-Basurto J., Rodríguez-Valdez L.M., Sánchez-Torres L.E., Nogueda-Torres B., Nevárez-Moorillón G.V. (2018). In vitro and in silico studies of terpenes, terpenoids and related compounds with larvicidal and pupaecidal activity against Culex quinquefasciatus Say (Diptera: Culicidae) Open Access. Chem. Cent. J..

[105] Luo Y., Egwolf B., Eric Walters D., Roux B. (2010). Ion Selectivity of α-Hemolysin with a β-Cyclodextrin Adapter. I. Single Ion Potential of Mean Force and Diffusion Coefficient. J. Phys. Chem. B.

[106] Seridi L., Boufelfel A. (2011). Simulations of docking C60 in β-Cyclodextrin. J. Mol. Liq..

[107] Hernández-Sánchez P., López-Miranda S., Guardiola L., Serrano-Martínez A., Gabaldón J.A., Nuñez-Delicado E. (2017). Optimization of a method for preparing solid complexes of essential clove oil with β-cyclodextrins. J. Sci. Food Agric..

